# An individual-based forest model links canopy dynamics and shade tolerances along a soil moisture gradient

**DOI:** 10.1098/rsos.150589

**Published:** 2016-02-10

**Authors:** Jean Liénard, Nikolay Strigul

**Affiliations:** Department of Mathematics and Statistics, School of Art and Sciences, Washington State University, Vancouver, WA, USA

**Keywords:** individual-based forest model, forest succession, shade tolerance, gap dynamics, crown competition, root competition, moisture gradient

## Abstract

Understanding how forested ecosystems respond to climatic changes is a challenging problem as forest self-organization occurs simultaneously across multiple scales. Here, we explore the hypothesis that soil water availability shapes above-ground competition and gap dynamics, and ultimately alters the dominance of shade tolerant and intolerant species along the moisture gradient. We adapt a spatially explicit individual-based model with simultaneous crown and root competitions. Simulations show that the transition from xeric to mesic soils is accompanied by an increase in shade-tolerant species similar to the patterns documented in the North American forests. This transition is accompanied by a change from water to sunlight competitions, and happens at three successive stages: (i) mostly water-limited parkland, (ii) simultaneously water- and sunlight-limited closed canopy forests featuring a very sparse understory, and (iii) mostly sunlight-limited forests with a populated understory. This pattern is caused by contrasting successional dynamics that favour either shade-tolerant or shade-intolerant species, depending on soil moisture and understory density. This work demonstrates that forest patterns along environmental gradients can emerge from spatial competition without physiological trade-offs between shade and growth tolerance. Mechanistic understanding of population processes involved in the forest–parkland–desert transition will improve our ability to explain species distributions and predict forest responses to climatic changes.

## 1. Introduction

Understanding and predicting of ecosystem changes under non-stationary disturbance regimes are some of the most challenging problems in ecology [1]. One of the major concerns is how climatic changes, in particular droughts, can affect forest structure and dynamics [2,3]. Forests can be considered as complex adaptive systems that employ emergent self-organization mechanisms across different spatial and temporal scales driven by individual competition and morphological plasticity to respond to forest disturbances and environmental changes [4]. The dynamics and spatial distribution of trees is tightly connected with different physiological traits and ecological tradeoffs, including shade and drought tolerances, growth rates and mortality under light and water limiting conditions [5–7]. Despite a very extended body of empirical data concerning different traits involved in shade and drought tolerance at the level of individual organisms [6,8–13], our understanding of particular mechanisms which drive ecosystem-level self-organization, forest succession, and the development of large-scale spatial patterns along soil moisture gradients is quite limited due to the difficulties in scaling up individual traits to the community level [5,11,14–16]. There are also substantial practical difficulties in designing long-term experimental studies on forest succession, related to planning and overall cost of experiments that require participation of several generations of scientists and non-stationarity of climatic variables over large time scales.

Modelling can be particularly useful in understanding forest self-organization, in particular, individual-based forest models allow for simulating self-organization processes that occur between individuals and up to the stand level [17–20]. Smith & Huston [21] have employed computer simulations to demonstrate that the existence of a negative trade-off between shade and drought tolerances could explain changes in forest succession patterns along soil moisture gradients. In particular, they have simulated effects of this trade-off between forest structure and dynamics using a spatially implicit individual-based forest model of the JABOVA-FORET family [17,19,22]. This fundamental trade-off assumption was formulated as follows: ‘Tolerances to conditions of low light and low moisture are interdependent and inversely correlated. Adaptations that allow a plant to grow at low light levels restrict its ability to survive under dry conditions. Conversely, adaptations that allow survival under dry conditions reduce the plant’s ability to grow in low light. Thus no woody plant can simultaneously have a high tolerance for low levels of both resources’ [21], p. 51, Premise 3. Computer simulations demonstrated that propagation of this trade-off to the community level results in the dominance of shade-intolerant trees in water-limited conditions, and dominance of shade-tolerant trees without water limitation. This attempt to mechanistically explain temporal and spatial forest patterns using computer simulations has received substantial attention and stimulated numerous experimental studies and analyses [7,12,23–31]. Overall the universality of this trade-off was not confirmed, and the premise posited in [21] has been highly criticized [10] (see also Discussion). At the same time, the decrease of shade-tolerant trees along the aridity gradient is well documented especially in North American forests [5,32]. The question then arises, are there mechanisms that do not involve this tolerance trade-off leading to the development of macroscopic shade tolerance patterns along moisture and latitude gradients?

In this study, we propose an alternative mechanism to explain the relatively higher dominance of shade-intolerant trees in arid conditions, which do not include physiological trade-offs between shade and drought tolerance. We focus in particular on gap dynamics, which refers to the processes of disturbance-driven mortality of canopy trees and their replacement by understory trees, that create a complicated patch mosaic of gaps (openings) in the forest canopy. Our hypothesis is that the observed patterns can be explained by different gap dynamics taking place on arid versus wet soils. Specifically, wet soils should permit a dense canopy and thus a shade-tolerant understory, while arid soils should result in a sparser overstory layer where shade-intolerant trees can get promoted. More generally, we hypothesize that forest macroscopic patterns can emerge through individual interactions alone, in particular through spatially explicit resource competition modified by environmental conditions.

## 2. Methods

### 2.1 Model overview

**Figure 1.**
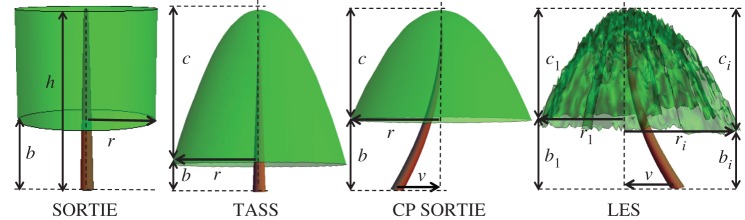
Crown shape representation in the spatially explicit forest simulator LES, and its predecessors SORTIE [18], TASS [34] and CP SORTIE [33]. SORTIE simulates the tree crown as a cylinder parametrized by the tree height *h*, bole height *b* and crown radius *r*. TASS model employs a complicated potential tree shape which has a cylindrical symmetry along the vertical line connecting the top of the crown and the root position. CP SORTIE also employs a radially symmetrical potential crown; however the vertical symmetry axis is determined by the centre of the crown position and the crown-vector *v* [35] reflects asymmetry caused by phototropism. The LES model simulates tree crown as a hierarchical structure consisting of 10×10 *cm* pixels representing small branches, sectors representing large branches (figure 3 and electronic supplementary material, figure S.2), and the crown-vector *v* [20]. Each sector *i* is characterized by its radius *r*_*i*_, living crown height *c*_*i*_ and bole height *b*_*i*_.

A spatially explicit, individual-based model called LES (named after the Russian word for forest) is employed to simulate spatial self-organization of forest stands [20]. The model represents a spatial stochastic process simulating development of each individual tree over its life cycle. The model is the next generation individual-based model in the JABOVA-FORET-SORTIE family [17,19,22]. The major novelties of the LES model [20] include tree below-ground competition and a new spatial algorithm of crown development, which improves crown representation in the crown plastic SORTIE (CP SORTIE) model [33] (figure 1).

In this work, the model is applied to simulate dynamics of a one-quarter hectare forest stand with a 1 year time step and 10×10 cm pixel resolution. Each forest stand simulation was conducted for *T*=1500 years and was initiated by a major disturbance event. The basic set of parameters describing tree above-ground and below-ground characteristics is presented in electronic supplementary material, table S1. Two prototypical species that have contrasting shade tolerance are modelled in this work to describe interplays between shade-tolerant and -intolerant species. The specific parametrizations are derived from white pine (*Pinus strobus*) and eastern hemlock (*Tsuga canadensis*). This two species model was intensively analysed before using the CP SORTIE model and the US Forest Inventory and Analysis dataset [5]. White pine is a faster growing tree species than eastern hemlock (linear growth coefficient *b*_1_ is 0.77134 versus 0.3498, cf. electronic supplementary material, table S1), while mortality of white pine in the understory is substantially larger than for eastern hemlock (20% versus 1%, cf. electronic supplementary material, table S1). These differences in growth–mortality rates result in the shade tolerance trade-off and early–late successional dynamics. The overall dynamics of this system can be represented by a macroscopic parameter called the shade tolerance index which represents the average value of trees’ shade tolerance in a forest stand, weighted by their basal area [32,36]. The shade tolerance index is computed using the formula

2.1 $$\mathrm{sti}=\sum_{t\in T}\frac{\rho_{t}\omega_{t}}{\sum_{t\in T}\omega_{t}},$$

where T is the set of trees in a stand, *ρ*_*t*_ are shade tolerance rankings of tree species and *ω* is the relative abundance metric. This parameter was studied in detail in several other publications [5,32,36] and it was shown that the estimates obtained with different abundance metrics (biomass, basal area and number of trees) are highly correlated [5].

**Figure 2.**
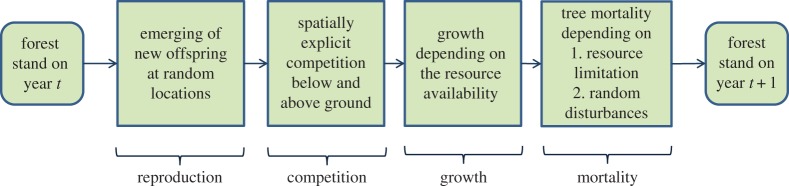
Annual transitions in the LES model.


The new model preserves substantial patterns from its predecessors, the SORTIE and CP SORTIE models [18,33–38]. In particular, the model employs the same general forest growth iteration process consisting of four steps (figure 2, pseudocode 1): spatial competition, growth, mortality and reproduction [33], fig. 5, p. 530. Like its predecessors, the model relies on tree dbh-height allometry and particular parametrizations [38], seedling emergence [33], mortality for shade-tolerant/intolerant species [39] and growth under simultaneous light and water limitations [37]. In the following, we describe processes occurring during each iteration focusing on the novel developments which distinguish this model from predecessors.

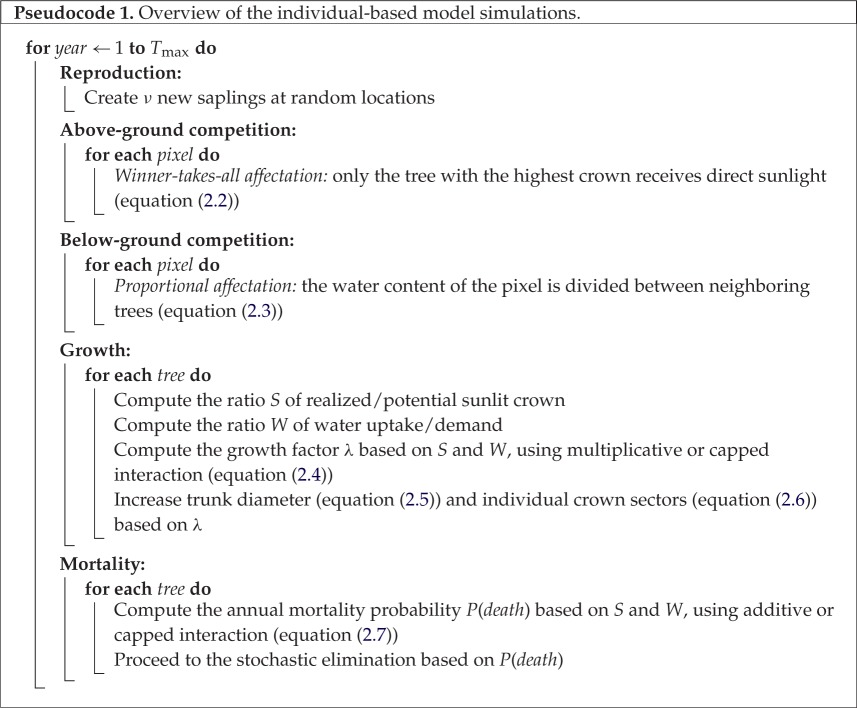


### 2.2 Competition for resources

**Figure 3.**
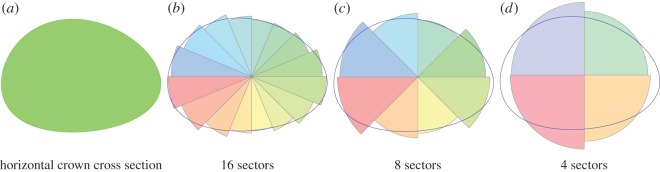
Crown approximation with 4, 8 and 16 sectors in the LES model. The LES model can operate with 2^*n*^ sectors with any integer *n*. The eight sector representation (*c*) provides a reasonably good balance between the discretization error and the computational resources required to simulate a several hectare forest stand. Models with 16 sectors (*b*) require too many computational resources, and ones with four sectors (*d*) do not satisfactorily approximate arbitrary crown shapes.

The major novelty of the LES model is the concurrent simulation of above- and below-ground competitions [20]. With respect to the crown competition process, the tree shape algorithm was substantially modified in order to relax the assumption of the radially symmetrical potential crowns. An additional motivation for the development of a more realistic crown structure is to enable crown shape parametrization in the LES model using three-dimensional reconstructions from remote sensing data [40,41] (and Discussion). The radial symmetry of potential crowns permitted running of spatially explicit forest stand simulations in SORTIE and CP SORTIE models (figure 1) using the limited computational resources of previous generation computers. However, this assumption was unrealistic as tree crowns restricted by spatial competition have symmetric potential crowns much larger than the actual crown (electronic supplementary material, figure S.1). Therefore, a tree released from competition (typically through the death of a neighbouring tree) was instantaneously gaining a substantial new part from the crown that altered its growth and survival. With respect to the stand-level dynamics this assumption often led to the instantaneous filling of canopy gaps. In the LES model, the crown of every tree is not radially symmetrical. Each crown is evenly subdivided into eight sectors representing large independently developing branches. The simulator allows computation of 2^*n*^ number of sectors for any integer *n*. Figure 3 illustrates the rationale for using the eight sector crown representation, as it is a good balance between computationally intense crown representations with a larger number of sectors and a large error in the area approximation when the number of sectors is smaller.

The shape of the sector *i* is determined by the sector radius *r*_*i*_, live crown ratio *β*_*i*_, tree height *h* and the species-specific crown shape parameter *C*. The live crown ratio is the crown height divided by the tree height (for instance, *β*_*i*_=*c*_*i*_/(*c*_*i*_+*b*_*i*_) in figure 1). The height of the crown at a distance *x*<*r*_*i*_ of the stem is computed using the crown shape profile formula [33], eqn 6, p. 528 applied to the particular sector *i*:

2.2 $$h=\left(1-\beta_{i}{\left(\frac{x}{r_{i}}\right)}^{\gamma }\right).$$

In this work, *γ*=0.5 for both species (electronic supplementary material, table S1) corresponding to the square-root crown shape profile [33], fig. 3, p. 528. The evolution of the crown representation along the model genealogy is presented in figure 1.

Similar to CP SORTIE model [33], equation (2.2) applied to all sectors determines the potential tree crown in a particular year. Then the spatial competition algorithm applied to the potential tree crowns produces the realized tree crowns, which are used for growth and mortality calculations. The set of sector radii {*r*_*i*_},*i*=1,…,8, is then updated where new radii are computed using the Mean estimate, which is less biased compared to the Min or Max estimates (electronic supplementary material, figure S.2).

We model the light capture using a ‘winner-takes-all’ attribution of the light available in each pixel, similar to the previous model [33]. In this scheme, only the tree whose shape has the highest altitude claim in a given pixel is able to capture a unit of sunlight. To determine trees receiving direct sunlight, we first loop over the pixels of the forest plots and then over the elevations claimed by each sector of each tree, breaking ties at random. The overall sunlight received by a canopy tree is finally expressed as the ratio *S* of light effectively received over the maximal crown extent (which corresponds to the potential light that would have been received in the absence of competition). In addition to the competition for direct sunlight, understory trees receive *c*_*t*_=9% of sunlight due to crown transparency [33,38].

The version of the LES model employed in this study has a slightly different root system representation from the version presented earlier [20]. In particular, in this study, trees develop their root systems in the circular zone in one soil level, while the previous version operates with root systems evolving independently in several sectors and three soil levels (see the electronic supplementary material, figure S.3 for the differences between this study and [20]). This model simplification allowed a substantial reduction in computer resource requirements. At the same time, our simulations demonstrate that this simplification did not affect the focal patterns of shade tolerance-driven species dynamics (figure 8).

**Figure 4.**
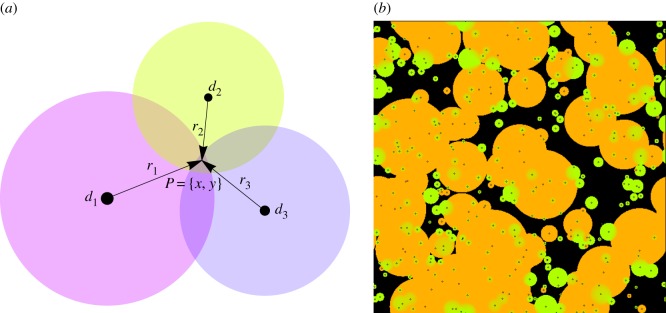
Root system competition in the LES model. (*a*) The focal point *P*=*x*,*y* is selected to illustrate the algorithm of tree competition. Positions of competing trees are indicated by black discs reflecting trunk diameters (*d*_1_, *d*_2_ and *d*_3_, respectively). The amount of water located at point *P* will be shared by these trees in proportion to their competition strength at *P* which is determined by trunk diameter *d*_*i*_ and by *r*_*i*_, the distance between tree and *P*. Overall, more water from a focal point *P* will be consumed by the tree with the highest competition strength (largest and closest to *p*). (*b*) Underground architecture of a simulated forest stand developing on soil with intermediate moisture regime (0.25 ha, 100 years after a major disturbance). Root systems are coloured with respect to the species (shade tolerant in green, shade intolerant in orange). With underground competition, several root systems can simultaneously extract water from the same spot (although their water uptake at that location is then reduced). In contrast with light competition, root systems overlap in space and small trees can have a root system entirely within a root system of a large tree.

Each root system is assumed to be circular in shape, with a diameter *d*_*r*_ expressed as a linear function of trunk diameter *d*: *d*_*r*_=*α* *d* [42]. In contrast to light allocation, several trees can simultaneously extract water from the same location (figure 4). Water is assumed to be a depletable resource, and the available water content of each pixel is divided according to below-ground competition. Specifically, each tree claims water with a strength *s* that is inversely proportional to the distance to the stem *x*_*r*_: *s*=1−*x*_*r*_/*d*_*r*_. The water content, *c*, of each pixel is then distributed so that the uptake of a specific tree *u*_*j*_ is the ratio between its own claims and the claims of the competing trees, summed over the whole extent of its root system:

2.3 $$u_{j}=\int_{\mathrm{root}\;\mathrm{system}}c\frac{s_{j}}{\sum_{k}s_{k}}.$$

Finally, the overall water uptake is expressed as the ratio, *W*, of the realized water uptake after the competition, *u*_*j*_, to the potential water demand computed for the given tree depending on its size, *d*_*r*_. Without competition with neighbours and when each pixel contains the base value of 1 unit of water, this ratio amounts to 1 and trees are not water limited; conversely, in the case of competition and/or of low water availability, the ratio becomes less than 1 and trees become water limited. Therefore, *W* depends on the soil water content, *c*, and below-ground spatial competition. The aridity index is defined as a [0−1] scale by varying the water content per unit, *c*, from 0 (where water is too scarce to allow any vegetation to grow) to 6 (where up to 6 trees can be simultaneously sustained on average). On this aridity scale, 0 corresponds to very xeric soils, while any index above 0.5 can be considered mesic.

### 2.3 Growth, mortality and reproduction

This version of the LES model employs the potential/realized growth modelling similar to previous models [33,20]. This general approach is modified to take into account growth limitation by both light and water. A tree’s realized growth is determined by potential growth (ideal growth) multiplied by the growth modifier, called the resource limitation factor *λ*. The difference between LES and the previous models is in the definition of *λ*, which is computed as a function of the light uptake ratio, *S* (i.e. the fraction of the realized to the potential crown), and the water uptake ratio, *W*. A number of different interactions have been hypothesized to account for the relationships between limiting resources, which differ depending on species [37,43]. In this study, we investigated two basic mechanisms underlying the interplay of resource limitations: (a) multiplicative interaction and (b) capped interaction where the scarcest resource limits the growth (Liebig’s law of the minimum):

2.4 $$\begin{array}{rl} \text{mechanism }\text{a}:\lambda & =SW\\ \mathrm{and}\;\text{mechanism }\text{b}:\lambda & =\min(S,W). \end{array}\}$$

Analogously to the CP SORTIE model [33], the trunk diameter *d* increases from year *y* to year *y*+1 according to the potential relative increment, *PRI*(*d*) (i.e. ideal growth increment computed for 1 year time step depending on tree dbh and species-specific constants *b*_1_, *b*_2_ and *b*_3_, cf. [44]), and the resource limitation factor *λ*, according to the formula

2.5 $$d^{\left(y+1\right)}=d^{\left(y\right)}+\lambda \mathrm{PRI}(d).$$

Similarly, each crown sector can expand given that there is no other tree to stop its progression. The radius *r*_*i*_ increment is then proportionally deduced from the diameter increment with a factor *ρ*:

2.6 $$r_{i}^{\left(y+1\right)}=r_{i}^{\left(y\right)}+\rho (d^{\left(y+1\right)}-d^{\left(y\right)}).$$

Mortality of trees is based on a stochastic random drawing based on resource limitation. Mortality induced by light limitation depends on the species’ shade tolerance and is included as a yearly probability *P*_*L*_ (estimated in accordance with [39], see the electronic supplementary material, table S1 for values). Mortality induced by water limitation is species independent and is taken as a probability $P_{W}=P_{\max}(1-W)$, where $P_{\max}$ reflects the maximal possible death probability due to water-limitation alone. We could not find consistent estimates of $P_{\max}$ in the literature, so we considered all values from 0 to 100% by increments of 10%; all values resulted in no overall qualitative difference as long as some mortality was associated with water limitation (i.e. $P_{\max}\neq 0\%$), and we settled with the minimal mortality of 10% for the results presented in the main text. We investigated two mechanisms that could account for the interplays between the mortalities induced by lack of light and water: (*α*) additive interaction and (*β*) capped interaction:

2.7 $$\begin{array}{rl} \text{mechanism}\;\alpha :P(\mathrm{death}) & =1-(1-P_{L})(1-P_{W})\\ \mathrm{and}\;\mathrm{mechanism}\;\beta :P(\mathrm{death}) & =\max(P_{L},P_{W}). \end{array}\}$$

The additive mortality *α* corresponds to the probability of surviving both sources of mortality, and is equal to or greater than the capped mortality *β* (which corresponds to the probability of surviving only the most threatening resource limitation).

As in previous models, new trees are generated as saplings with a random probability of appearance at any pixel, and with a diameter *d* randomly chosen between 3 and 5 cm. In this work, we do not simulate trade-offs associated with density-dependent fecundity [33]. The parameter *ν* constrains the number of new saplings appearing every year, set to 40 new sapling per hectare.

## 3. Results

### 3.1 Sensitivity analysis

**Figure 5.**
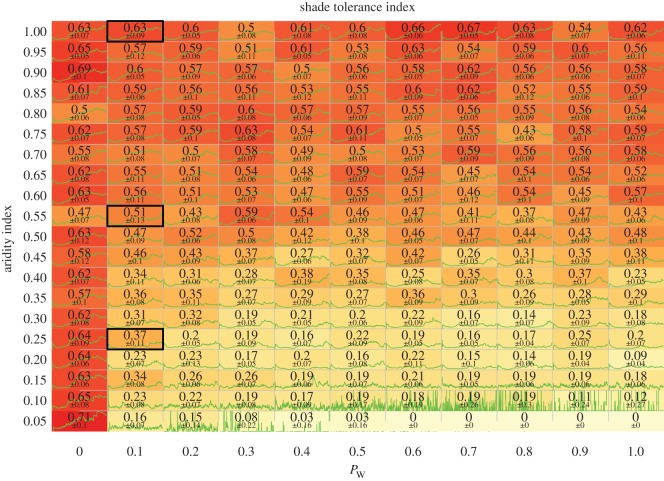
Shade tolerance index as a function of *P*_W_, the mortality induced by water limitation (*x*-axis), and as a function of the aridity index (*y*-axis). Inside each cell is displayed the mean shade tolerance index at equilibrium ± its standard deviation. In addition, the green curve inside each cell shows the dynamics of the shade tolerance for 750 years following a complete disturbance. The three cells corresponding to the particular simulation parametrizations displayed in main text (figure 7) are framed by a thick black line. A wide variability of shade tolerance index is observed for low aridity indices (less than 0.15), as a result of very sparse forests with a quick turnover rate (with also almost no basal area, see the electronic supplementary material, figures S.12 and S.13). The combination of mechanisms shown here is the ‘Liebig’s law’ mortality coupled with multiplicative growth; however, similar results are obtained for all combinations as the simulations are robust to change in mechanisms (see electronic supplementary material, figures S.4–S.13).

While most simulation parameters can be set according to the literature (electronic supplementary material, table S1), we could not find reliable estimates for *P*_W_, the mortality induced by water limitation. We thus conducted a sensitivity analysis by varying this parameter from 0 to 100%, by steps of 10% (with an extra step at 5%, cf. figure 5). All stand-level characteristics (including mean age, shade-tolerance index, basal area and understory/overstory composition) were found to exhibit highly similar patterns for non-null mortality induced by water limitation (*P*_W_≥5%). In addition, using different mechanisms for growth (additive/Liebig’s law, cf. equation (2.4)) and mortality (multiplicative/Liebig’s law, cf. equation (2.7)) resulted in similar patterns for all characteristics: the shade tolerance index (electronic supplementary material, figure S.4), the basal area (electronic supplementary material, figures S.5 and S.6), the proportion of shade-tolerant/intolerant trees (electronic supplementary material, figures S.7 and S.8), the mean age (electronic supplementary material, figure S.9), the crown symmetry ratio (electronic supplementary material, figure S.10), the canopy cover (electronic supplementary material, figure S.11) and the number of trees per hectare (electronic supplementary material, figures S.12 and S.13). This overall shows the robustness of the model with respect to different mechanisms of growth and mortality. In the following, we investigate the patterns of stand-level characteristics with respect to soil aridity. We set *P*_W_=20% for the analysis in the main text; similar patterns are observed for other values (electronic supplementary material, figures S.14–S.17 use *P*_W_ of 5, 10, 30 and 40%).

### 3.2 Overall effects of water limitation on forest structure

**Figure 6.**
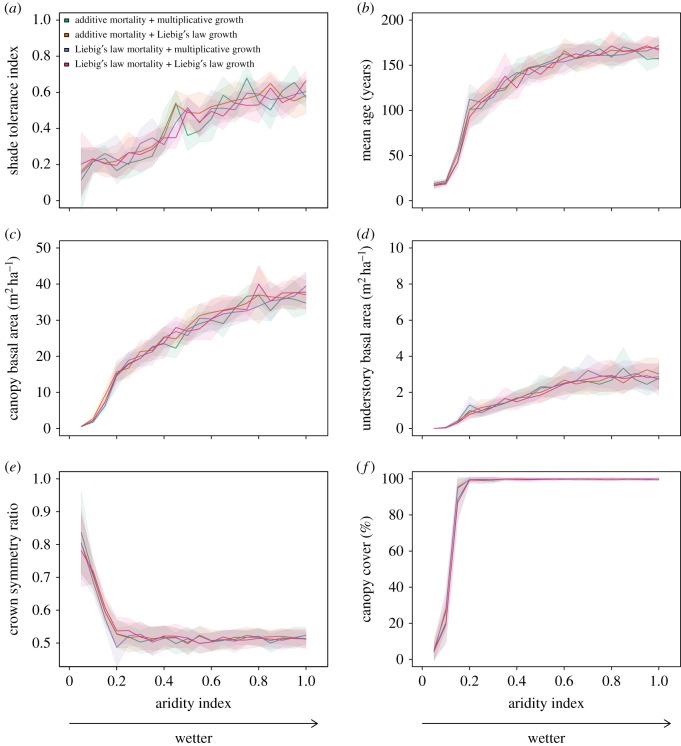
Stand characteristics at equilibrium for different soil aridity indices, from 0 (xeric) up to 1 (mesic). Simulations relied on different mechanisms for mortality (additive or Liebig’s law) and growth (multiplicative or Liebig’s law). Shaded areas display standard deviations of values. (*a*) Shade tolerance index, (*b*) average age of trees, (*c*) basal area of canopy trees, (*d*) basal area of understory trees, (*e*) crown symmetry ratio and (*f*) canopy cover (forest floor proportion covered by tree crowns vertical projection).

We computed the equilibrium of stand-level characteristics by averaging their values for the second half of the 1500-year simulations. Not surprisingly, xeric soils were found to be less suitable to sustain vegetation than mesic soils. In particular, drier conditions were associated with lower mean age of trees (figure 6*b*), lower basal area (figure 6*c*,*d*) and lower canopy cover (figure 6*f*). In addition, a clear pattern of increasing shade tolerance index was apparent with more mesic soils (figure 6*a*) resulting from a relative increase in shade-tolerant species compared with shade-intolerant species. The increase in soil moisture was further accompanied by a progression from circular to more oval tree crowns (figure 6*e*), thus revealing higher degrees of light competition in mesic soils.

### 3.3 Interplays between water and sunlight limitations

In the simulations, canopy cover was found to increase with soil moisture, reaching a closed canopy for aridity indices as low as 0.2 (figure 6*f*). The switch from water to sunlight limitation is reflected in the progression towards highly assymetric crown shapes (up to aridity indices of about 0.2, figure 6*e*). This transition from parklands to forests marks systematic competition for sunlight and is a turning point of an ecosystem. In conditions drier than this point, the ecosystem is mostly limited by water, and the shade tolerance index is stable (figure 6*a*). After the transition, with aridity indices in the range of 0.2–0.8, sunlight and water are co-limiting factors in closed canopy forests. In this range of aridity indices, basal area and stand age show sustained increases as soil moisture conditions become more favourable to individual trees (figure 6*b*–*d*). The switch from water to sunlight competition allows a more populated understory (figure 6*d*) and enables shade tolerance to be a more important driver of succession (reflected in the increase in shade tolerance index when aridity index is in [0.2,0.8], in figure 6*a*). With aridity indices higher than 0.8, sunlight acts as the most important limiting factor on forests. At this point, more soil moisture becomes irrelevant, which results in stable values for all stand-level characteristics (figure 6*a*–*f*).

We also conducted a sensitivity analysis of species parameters in order to evaluate how general the results are over a wide range of tree growth and mortality parameters. In these simulations, each tree was given an original intermediate parametrization. Formally, this intermediate parametrization is a linear combination of parameter vectors of white pine and eastern hemlock presented in electronic supplementary material, table S1. In particular, a tree parameter vector *p*(*α*) is defined as *p*(*α*)=*αp*(0)+(1−*α*)*p*(1), where *α*∈]0,1[, and *p*(0) and *p*(1) are parameter vectors of white pine and eastern hemlock, respectively. For example, *α*=0.5 results in a parametrization halfway between the two prototypical species with a shade tolerance rank of 0.5. The random variable *α* was sampled using the polynomial mutation operator derived from multi-objective optimization theory [45], and used with a distribution index *η*_*m*_=5 [46]. The use of this operator to sample the [0,1] space results in a symmetrical distribution with two centres close to the original parametrizations of white pine and eastern hemlock (the centres are located at *α*_*WP*_=0.05 and *α*_*EH*_=0.95). The results of these simulations are reported in the electronic supplementary material, figure S.18. We found that the model is robust to variations in species growth and mortality parameters within our domain defined by the values presented in electronic supplementary material, table S1. The simulations demonstrate the same progression of shade tolerance index from xeric to mesic soils, as reported in figure 6.

### 3.4 Competition dynamics over time

**Figure 7.**
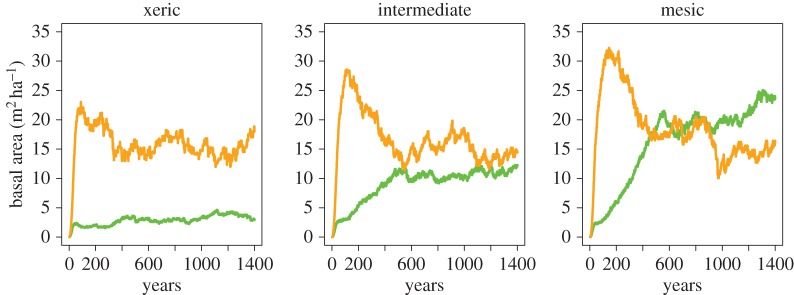
Dynamics of shade-tolerant species (green) and shade-intolerant species (orange) as a function of time since last disturbance, for three different soil moistures.

**Figure 8.**
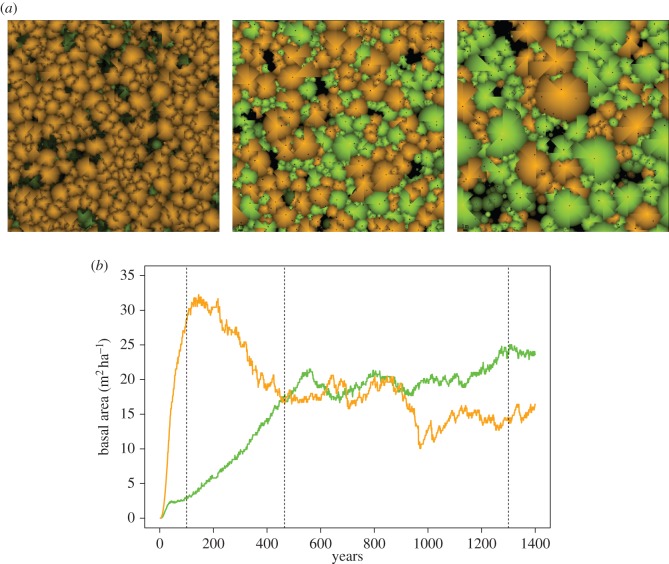
Three snapshots from above a 1 ha simulation (*a*) taken at different years after a complete disturbance. Shade-tolerant trees are displayed in green and shade-intolerant species are displayed in orange. The graph (*b*) shows the basal area of species across time; dashed vertical lines indicate the times at which the snapshots were taken.

We also studied dynamics following a simulated clear-cut. In the case of mesic soils, we observe replacement patterns in full accordance with the classic paradigm of shade tolerance-driven succession (e.g. [5]). Specifically, we observe an early domination of shade-intolerant trees for 150 years, followed by competition by shade-tolerant trees spanning until about 500 years, and eventually a stable higher prevalence of shade tolerance trees (figures 7 and 8). In the case of xeric and intermediate soils, the species dynamics is substantially different (figure 7). The early rise of shade-intolerant species during stand initiation is clear, consistent with their faster growth capacities. However, understory reinitiation remains minimal in xeric and intermediate soils as the available water is collected by already existing trees. As a consequence, the late domination of shade-tolerant trees is prevented when soil moisture is limited.

## 4. Discussion

Light and water are major factors in the development of individual plants and landscape patterns alike. However, our ability to explain changes occurring in various site conditions from individual traits is limited. We investigated the connection between shade tolerance patterns and alterations of forest gap dynamics along the water availability gradient. A spatially explicit individual-based model that integrates water and sunlight competitions was employed to simulate a desert–savannah–forest transition. The model simulates the spatially explicit interactions of shade-tolerant and -intolerant trees using parameters of two North American tree species eastern white pine (*Pinus strobus*) and eastern hemlock (*Tsuga canadensis*). The transition is accompanied by change from water to sunlight competitions, and happens at three successive states: (i) mostly water-limited parkland, (ii) simultaneously water- and sunlight-limited closed canopy forests featuring a very sparse understory, and (iii) mostly sunlight-limited forests with a populated understory. We demonstrate that changes in the overstory canopy from relatively open to closed due to increased soil moisture could explain the dynamics of shade-tolerant versus shade-intolerant species. Our study is the first theoretical account of this phenomenon. This work further demonstrates that altered gap dynamics is sufficient to explain the empirically observed increase in shade tolerance across a soil moisture gradient [32].

### 4.1 Towards a new generation of individual-based forest simulators

In this study, we present a new generation, individual-based forest model capable of incorporating above- and below-ground competitions. Linking processes across scales is difficult because we currently lack an integrated theoretical framework describing self-organization in forest ecosystems as well as the quantitative framework for implementing the theory [47–49]. Numerous distinct processes occur simultaneously at the genome, cell, organ, individual organism, population and landscape levels of forest organization. Gene expression and cellular processes occur at spatial and temporal scales of micrometres and milliseconds. Individual tree growth and competition for resources can be studied at scales measured in the ranges of centimetres–metres and months–years, while processes at the ecosystem level, such as forest succession, occur on scales of kilometres and decades. Individual-based forest simulators have been employed in forest ecology since the 1960s to forecast forest dynamics by simulating tree life cycle and spatial competition, allowing researchers to model effects of ecological trade-offs and predict forest dynamics at intermediate scales [17–19,33,38,50–52]. Individual-based models of the earlier generation were spatially implicit, and thus limited in their simulation of gap dynamics [17,19,21,22]; the next generation of spatially explicit models has been able to efficiently capture forest gap dynamics and succession driven by the shade tolerance trade-off [18,33,38,51,53,54]. The model presented in this paper simulates tree morphological plasticity and underground competition concurrently, and enables the study of their interactions scaled to the community level for the first time.

The robustness of model predictions to their assumptions and specific parametrization is a long-standing concern [50,51]. This issue is further related to model complexity, and grows with the inclusion of each additional mechanism. Compared to its predecessors, the most important change in LES is the simultaneous above- and below-ground competition simulations. Here we conducted numerous stochastic simulations in order to calibrate the model and assess its sensitivity with respect to a broad range of assumptions concerning individual tree physiology. In particular, we tested several alternative mechanisms of joint light and water limitations and different parameter values as well. In addition, we verified that the results hold true when using the cylindrical crown shape employed by SORTIE. The consistent robustness of model predictions to these changes demonstrates that the increase in relative abundance of shade-tolerant trees accompanying the increase in soil water availability might be a universal characteristic and can possibly be applied broadly to different ecosystems.

The detailed three-dimensional geometric representation of each individual tree crown in the LES model can be directly linked to the remote sensing data. In particular, it is possible to use airborne LIDAR data to estimate some spatial characteristics of individual tree crowns from nadir LIDAR measurements [55]. More detailed and precise three-dimensional crown reconstructions can be obtained with ground-based LIDAR or unmanned aerial vehicle-based photogrammetry [40,41]. These recently developed three-dimensional reconstruction methods allow for precisely estimating spatial crown characteristics and tree coordinates. Precise three-dimensional reconstructions can be further employed for spatially explicit simulations of particular forest stands with the LES model.

### 4.2 Understanding of forest self-organization and emergence of macroscopic patterns

Debates in forest ecology on the role of shade and drought tolerances in forested ecosystems have been focused on how physiological trade-offs can contribute to the development of community-level patterns [6,7,11,56,57], while the present work is focused on the role of the gap dynamics. The two hypotheses relating drought and shade tolerance are usually in opposition [26–28]. The *trade-off hypothesis* states that shade tolerance is inversely proportional to drought tolerance [21]. The *facilitation hypothesis* posits that shade tolerance is proportional to drought tolerance [23]. Intermediate views emerge from meta-analyses, with species in the Northern Hemisphere being generally consistent with the trade-off hypothesis but also seldom exhibiting simultaneous tolerance to several stressors [12]. It is also a possibility that facilitation and trade-off occur at different ends of the irradiance spectrum [27]. Consistent with this, experiments on seedlings offer a nuanced vision depending on the species and irradiance levels studied, with observations in favour of the trade-off hypothesis [29,30] and others against it [7]. In this study, we did not assume any physiological difference between shade-tolerant and -intolerant species related to drought, as our goal was to specifically study interactions at the community level independent of these physiological trade-offs. Gap dynamics can thus be seen as a complement to physiological trade-offs, both of them contributing to shade tolerance changes along soil moisture gradients.

Our model predicts increases in understory and overstory tree abundance with greater water availability, in agreement with a broad range of experimental data and field observations [16,11]. It is also well known that the moisture gradient affects the species distribution [58–62]. A more specific prediction of our model concerns the relative importance of shade-tolerant/intolerant species, and the corresponding increase in shade tolerance index. This model prediction is also in agreement with recently published statistical analyses of the USA and Quebec forest inventories [32,63]. Specifically, shade tolerance is linked with soil moisture in North American temperate and boreal forests [32], and the shade tolerance index increases along soil moisture gradients in different ecoregions. This increase was significant in most North American biomes, except for three subtropical/tropical ecoregions of the southeastern USA [32]. The current simulations reinforce the previously observed relationship between shade tolerance and soil moisture with temperate species, while providing a possible mechanism for the development of these patterns.

## Supplementary Material

Appendix. Sector representation of the tree crown in the LES model and calibration of the mortality rate depending on water limitation and growth/mortality mechanisms Table T1. Parameters used in computer simulation
