# Supplementary material for: An individual-based forest model links canopy dynamics and shade tolerances along a soil moisture gradient
Source: R Soc Open Sci. 2016 Feb 10;3(2):150589. doi: 10.1098/rsos.150589 (PMC4785980; doi:10.1098/rsos.150589)
Supplement: Appendix. Sector representation of the tree crown in the LES model and calibration of the mortality rate depending on water limitation and growth/mortality mechanisms Table T1. Parameters used in computer simulation [file rsos150589supp1.pdf]

9. Appendix

Parameter	Symbol	Value	Dimension	Reference
Tree geometry and demography				
Root/diameter growth ratio	α	20	ND	A
Crown/diameter growth ratio	ρ	15	ND	B
Crown profile constant	γ	1/2	ND	C
Fecundity constant (new saplings per year)	ν	40	#/year	C
Initial sapling dbh	d_i	3-5	cm	C
Initial live crown ratio	β	90	%	C
Overstory random disturbance rate	μ_O	1	%	D
Stand simulation and canopy characteristics				
Crown transparency	c_t	9	%	CD
Stand area in simulations		1/4	ha	
Pixel resolution in simulations		10	cm^2	
Time step in simulations		1	year	
Simulation period	$T_{\max}$	1500	year	
Eastern hemlock (*Tsuga canadensis*) parameters				
Understory random disturbance rate	μ_U	1	%	E
Potential relative increment (PRI) values	b_1	0.3498	ND	F
	b_2	-0.436216	ND	F
	b_3	0.968043	ND	F
White pine (*Pinus strobus*) parameters				
Understory random disturbance rate	μ_U	20	%	E
Potential relative increment (PRI) values	b_1	0.77134	ND	F
	b_2	-0.5539	ND	F
	b_3	0.9797	ND	F

A:[28] B:[5] C:[60] D:[48] E:[37] F:[9]

Table 1: Parameters used in computer simulation. ND indicates that the quantity is dimensionless.

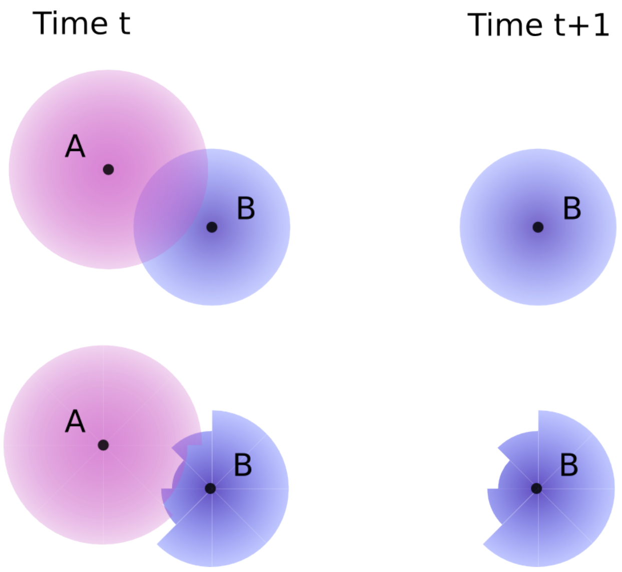

Figure S.1: Comparison of radially symmetrical potential crown shape models (CP SORTIE and its predecessors, at the top) and the modular sector structure (LES model, at the bottom) following the death of a neighboring tree with overlapping crown. In this illustration, the tree A (in purple) dies between the two time steps (t and $t+1$). In the model with radially symmetrical potential crown (CP SORTIE model), the realized crowns of trees competing for light is determined by a tessellation algorithm at time t. After the tree A dies, at time $t+1$ the realized crown of the surviving tree B (in blue) extends instantaneously to its potential maximal radius (determined by its height and d.b.h.). In the modular crown model (LES model) illustrated at the bottom, the tree shape consists of 8 sectors representing independently developing large branches. In this case, after the death of the tree A, the crown of the tree B does not change instantaneously.

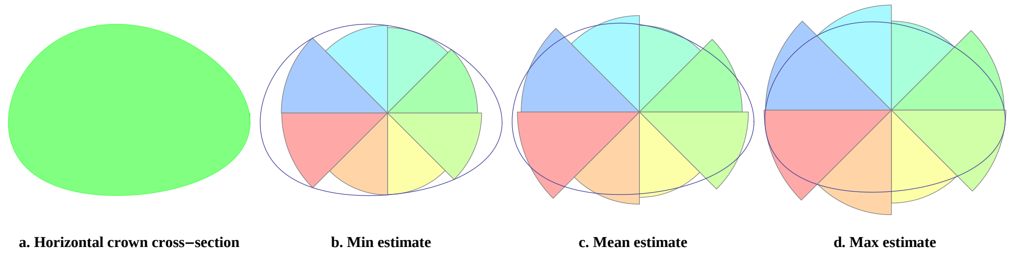

Figure S.2: Alternative estimates of the sector radius from realized tree crown cross-section. Horizontal cross-section of an arbitrary crown (a.) is approximated by 8 independent sectors using different estimates for the sector radius from the center of the crown: the min rule (b.) calculated the minimal distance, the mean rule (c.) estimates the average distance to all boundary points of the crown within the given sector, the max rule (d.) estimates the maximum distance. The mean rule was implemented in the LES model as it has the least bias, however this rule is more computationally intensive than the alternative rules.

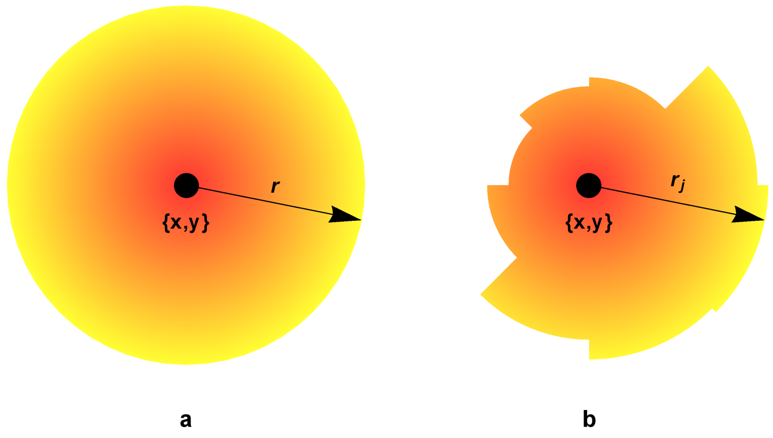

Figure S.3: Root system representation adapted in the current version of the LES model (a) and the original algorithm (b) [59]. The black disk located at the point with x, y coordinates illustrates the location of the tree base and the trunk diameter. The color gradient illustrates that the root density and the strength of root competition decreases with distance from the tree base. In the simulations presented the root system has radial symmetry (a). The original algorithm (b) employed a modular structure where sectors represents independently developing large roots. A large set of preliminary simulations demonstrated that the modular root structure does not substantially affect the transition from mesic to xeric soils, but is computationally very demanding. The adopted symmetrical root system (a) allowed for substantial reduction in computational load.

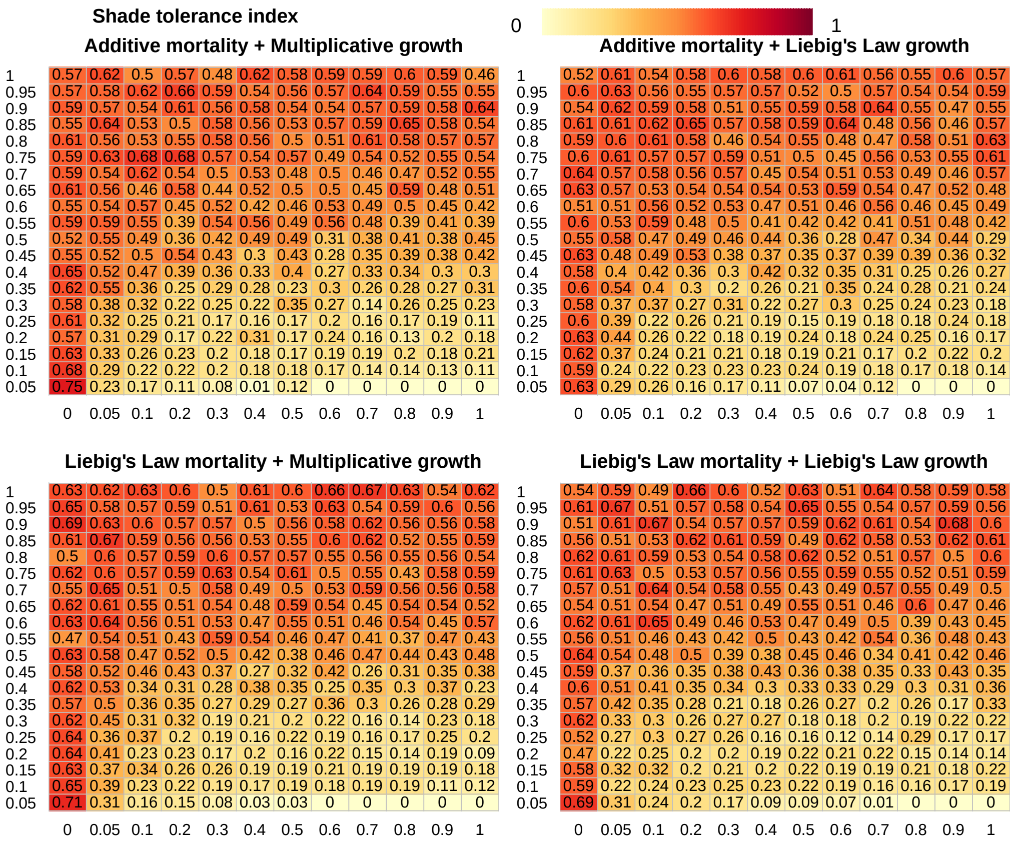

Figure S.4: Shade tolerance index at equilibrium as a function of P_W, the mortality induced by water limitation (x-axis) and as a function of the aridity index (y-axis). The different combinations of mechanisms (Additive or Liebig's Law mortality with Multiplicative or Liebig's Law growth) lead to similar patterns, thus showing robust effects of simulations.

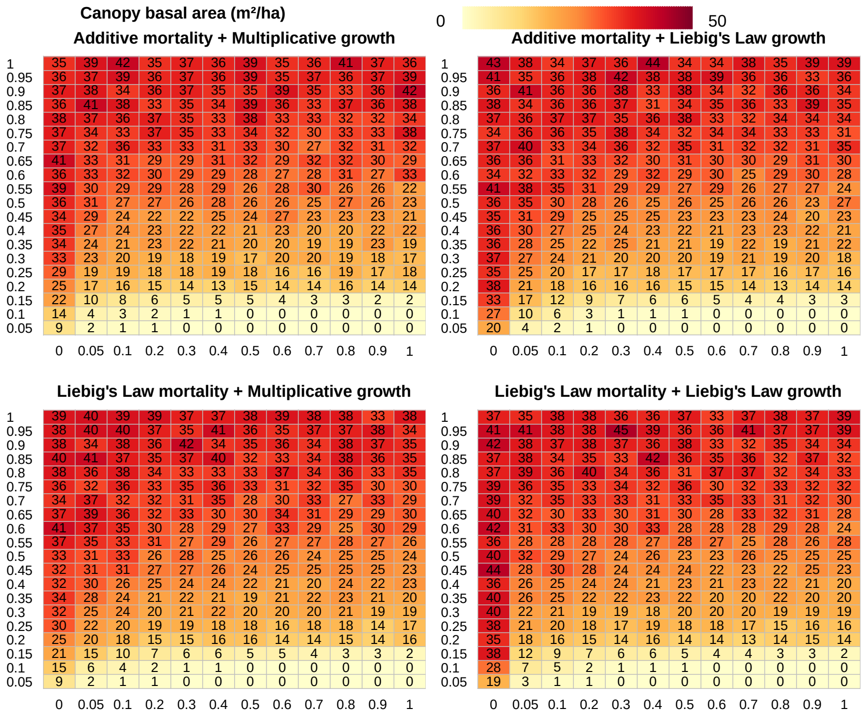

Figure S.5: Basal area of canopy trees at equilibrium for all studied mechanisms. Please refer to Fig. S.4 for notations.

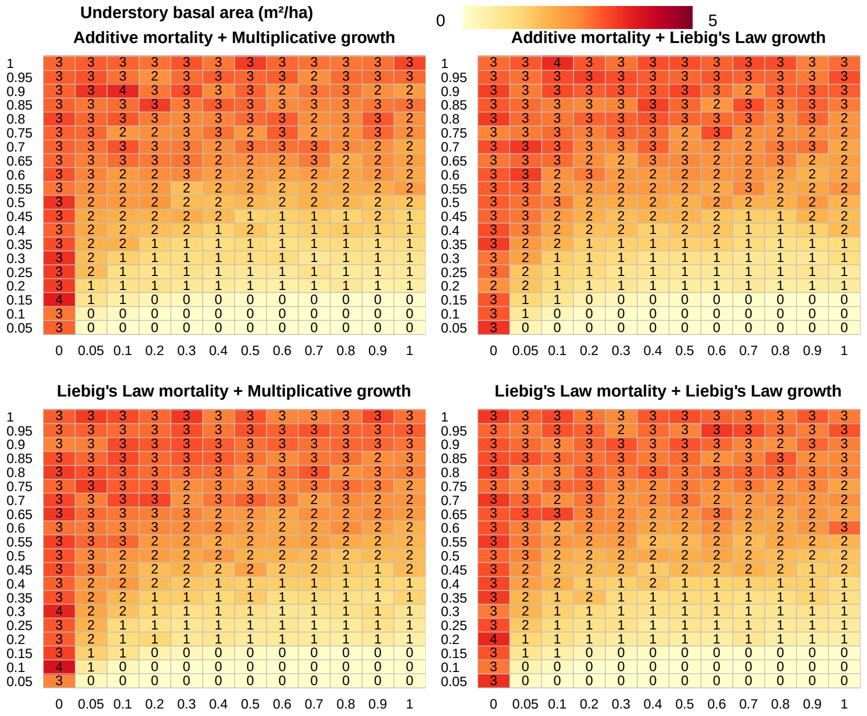

Figure S.6: Basal area of understory trees at equilibrium for all studied mechanisms. Please refer to Fig. S.4 for notations.

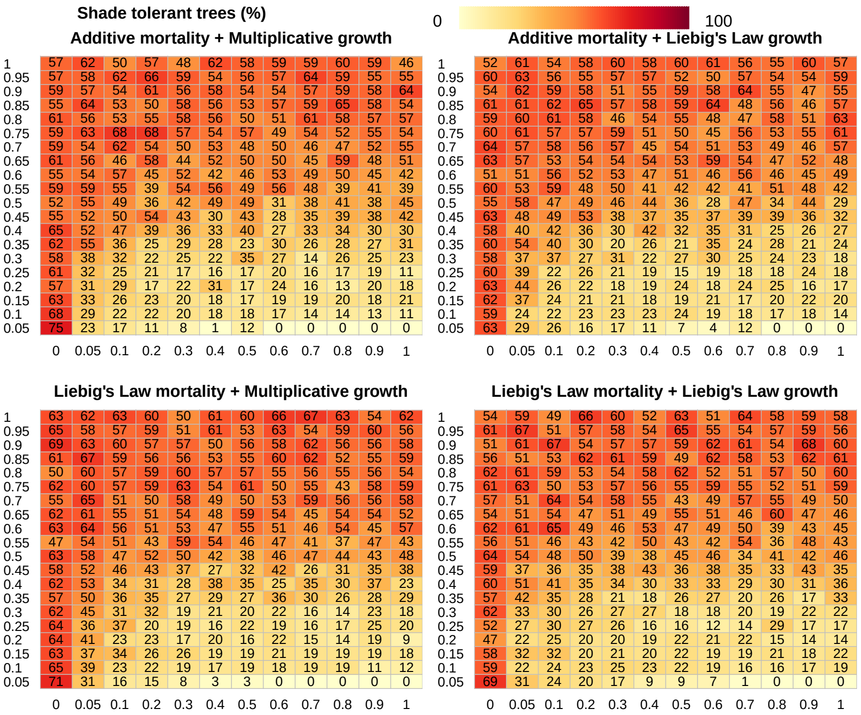

Figure S.7: Proportion of shade tolerant trees at equilibrium for all studied mechanisms. Please refer to Fig. S.4 for notations.

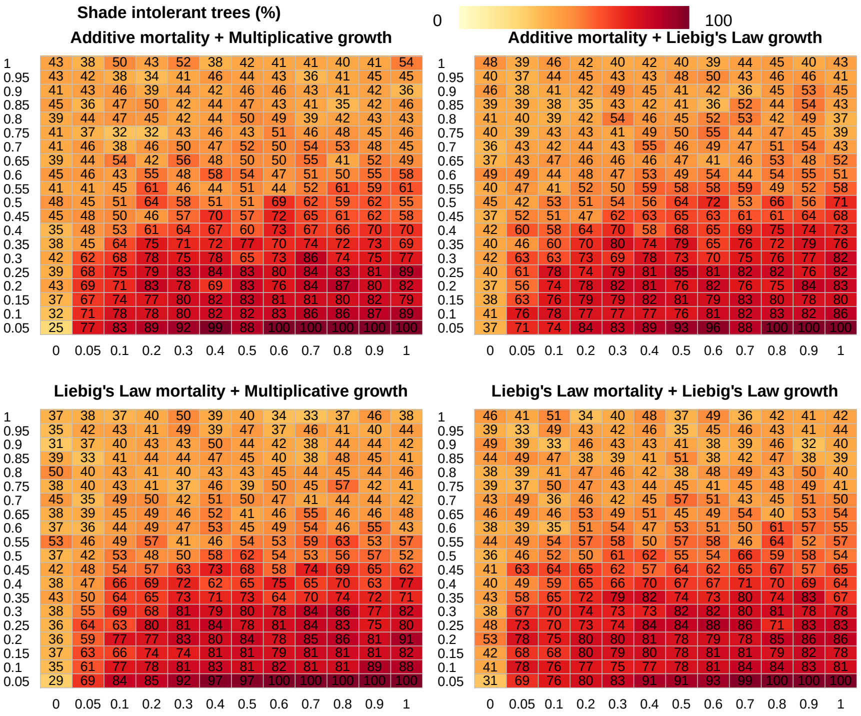

Figure S.8: Proportion of shade intolerant trees at equilibrium for all studied mechanisms. Please refer to Fig. S.4 for notations.

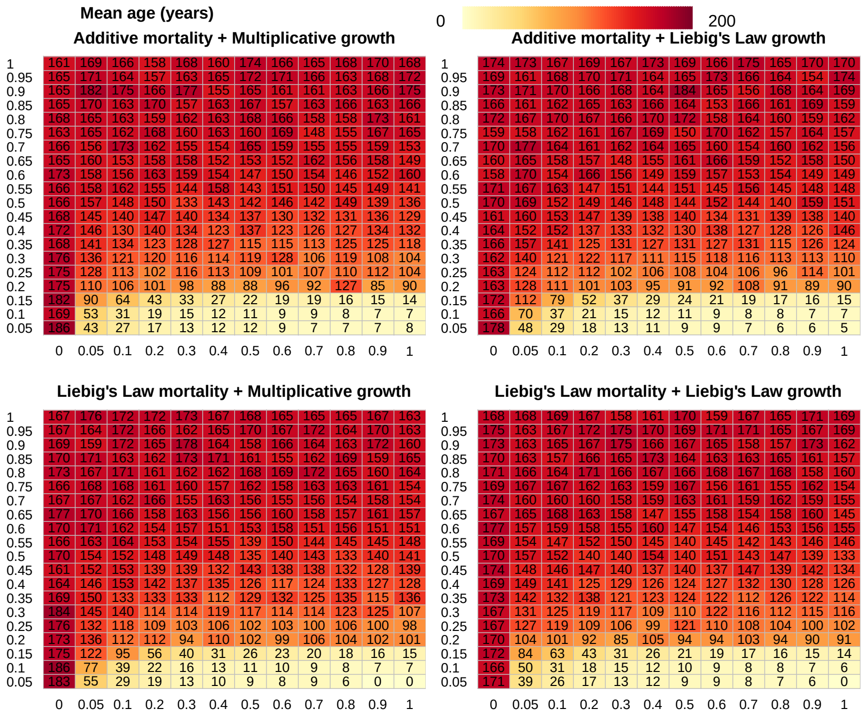

Figure S.9: Mean age at equilibrium for all studied mechanisms. Please refer to Fig. S.4 for notations.

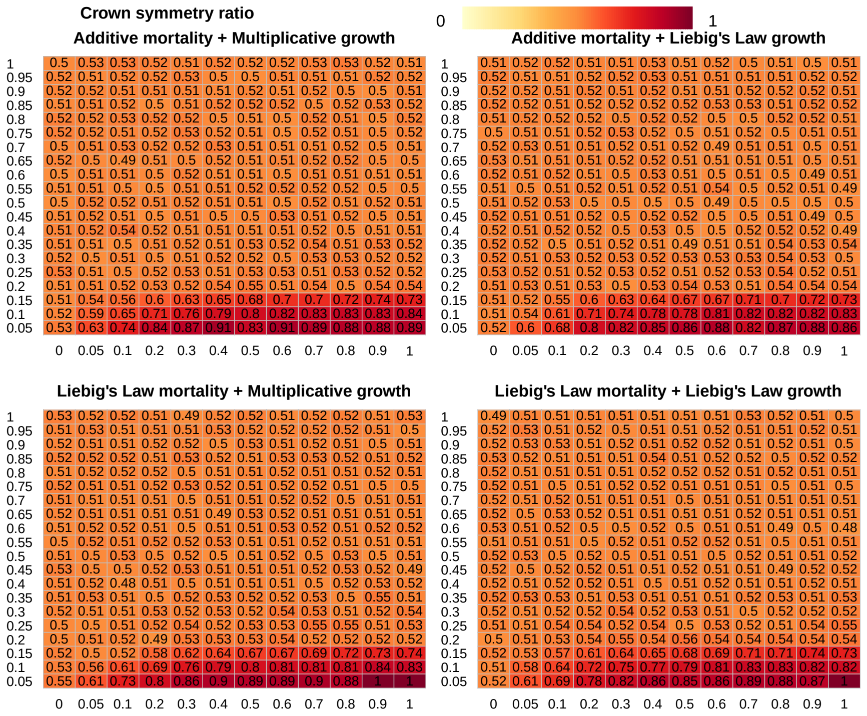

Figure S.10: Crown assymetry ratio at equilibrium for all studied mechanisms (1 being a perfectly circular crown). Please refer to Fig. S.4 for notations.

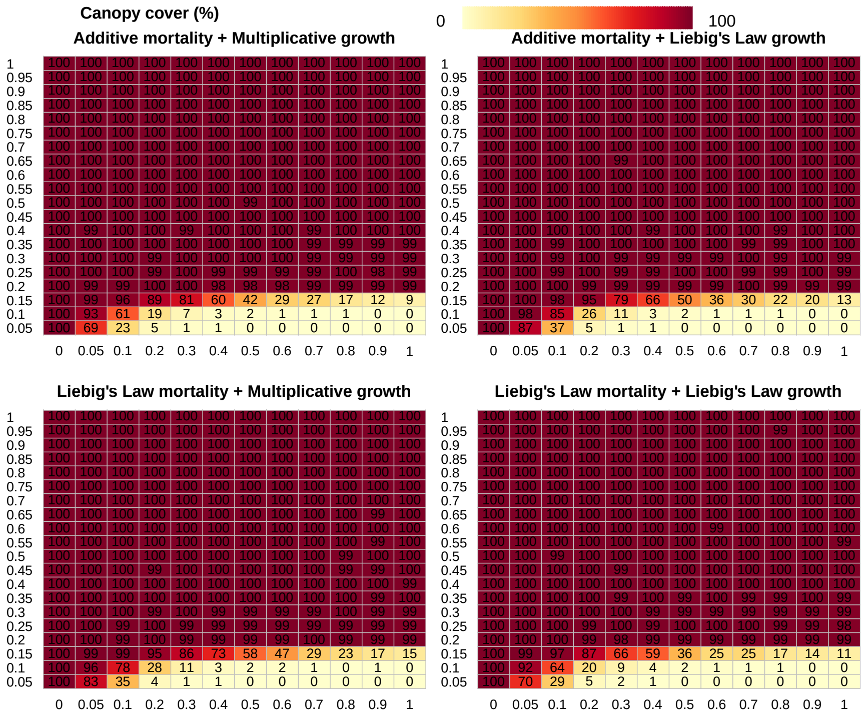

Figure S.11: Canopy cover at equilibrium for all studied mechanisms. Please refer to Fig. S.4 for notations.

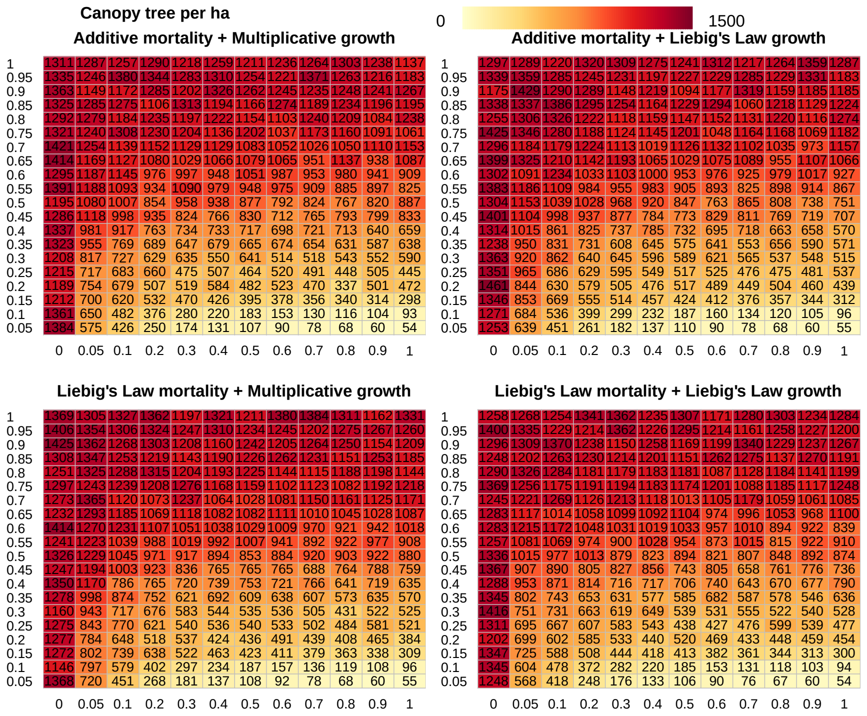

Figure S.12: Number of canopy trees at equilibrium for all studied mechanisms. Please refer to Fig. S.4 for notations.

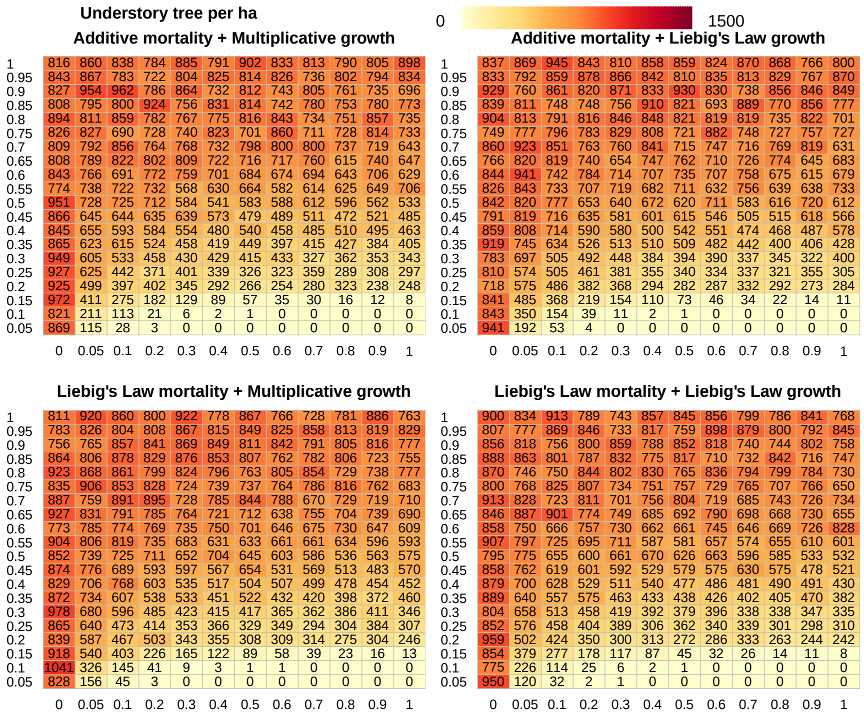

Figure S.13: Number of understory trees at equilibrium for all studied mechanisms. Please refer to Fig. S.4 for notations.

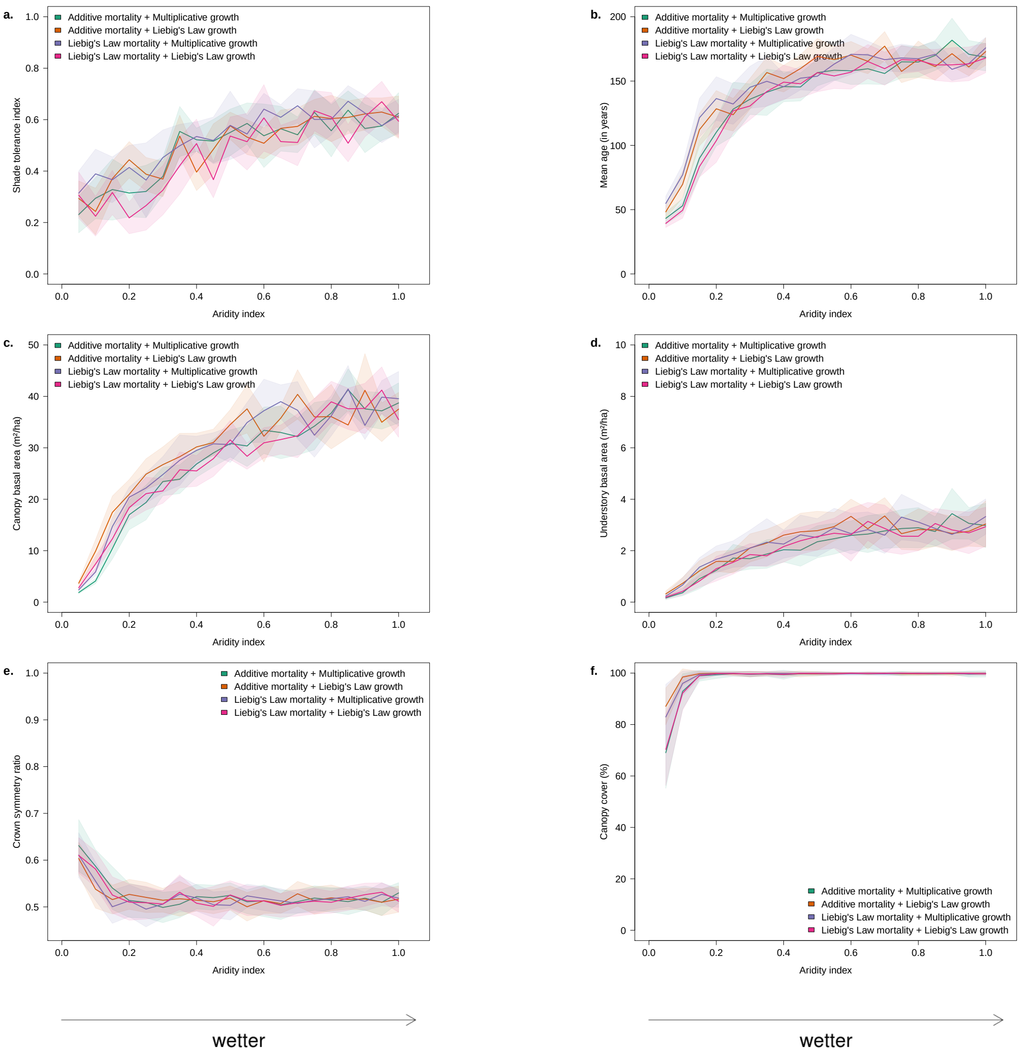

Figure S.14: Stand characteristics at equilibrium for different soil aridity indices, from 0 (xeric) up to 1 (mesic). Similar to Fig. 6 in main text, except that the maximal mortality level for water-limited trees was 5%.

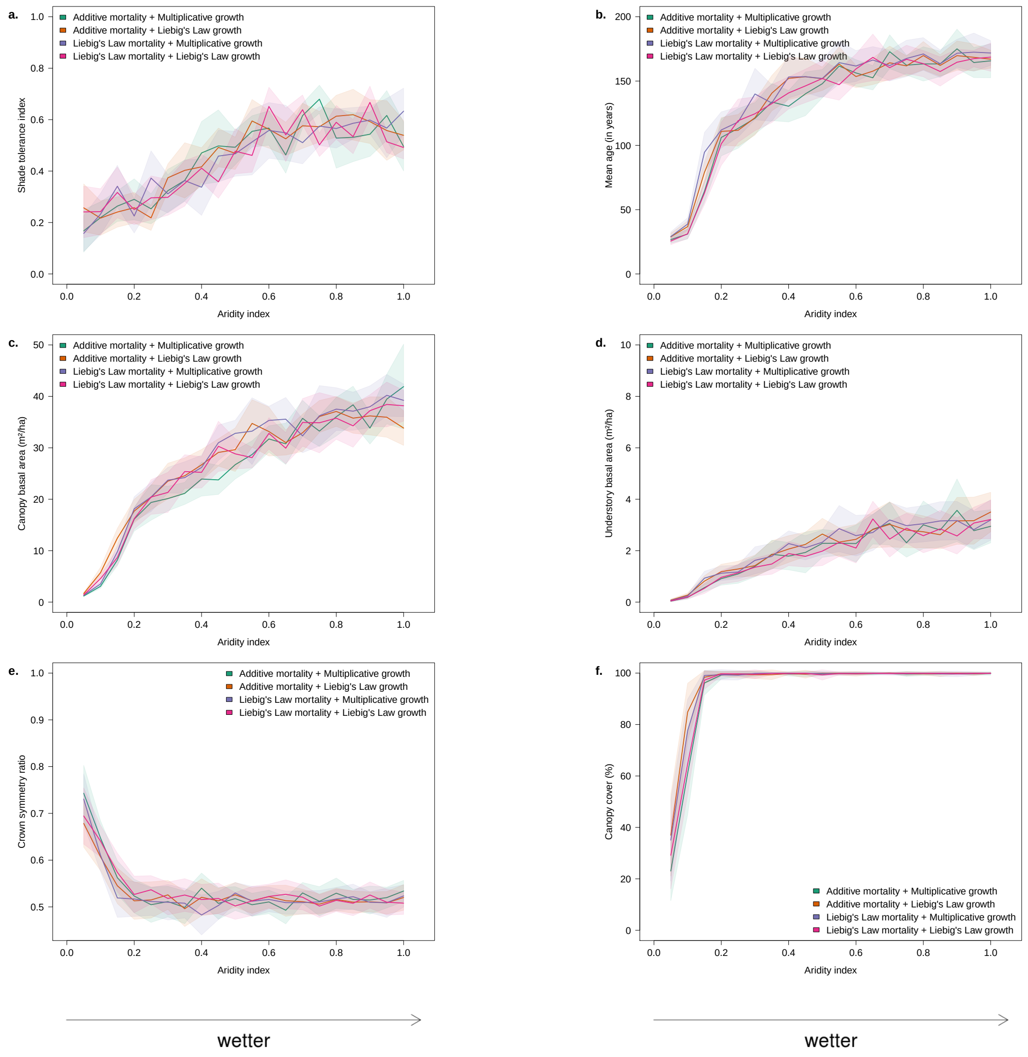

Figure S.15: Stand characteristics at equilibrium for different soil aridity indices, from 0 (xeric) up to 1 (mesic). Similar to Fig. 6 in main text, except that the maximal mortality level for water-limited trees was 10%.

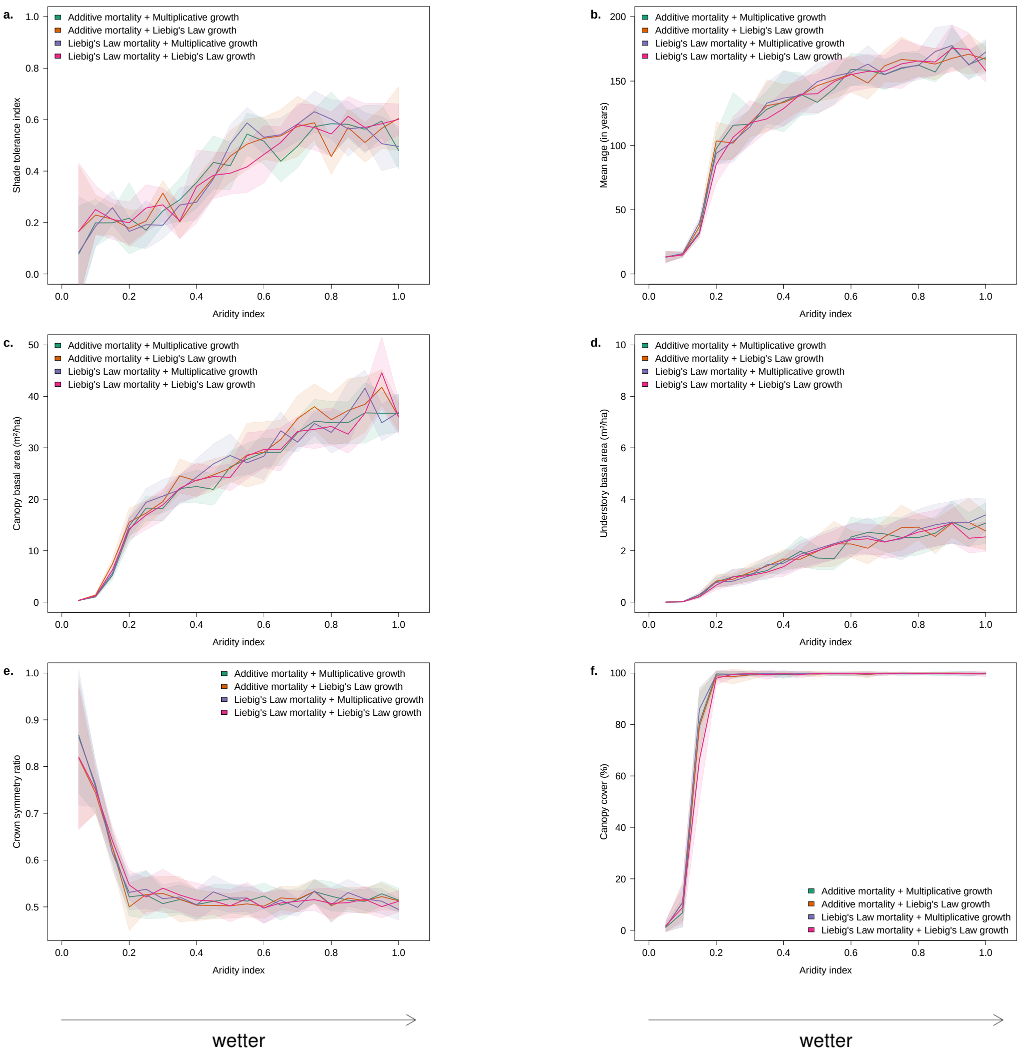

Figure S.16: Stand characteristics at equilibrium for different soil aridity indices, from 0 (xeric) up to 1 (mesic). Similar to Fig. 6 in main text, except that the maximal mortality level for water-limited trees was 30%.

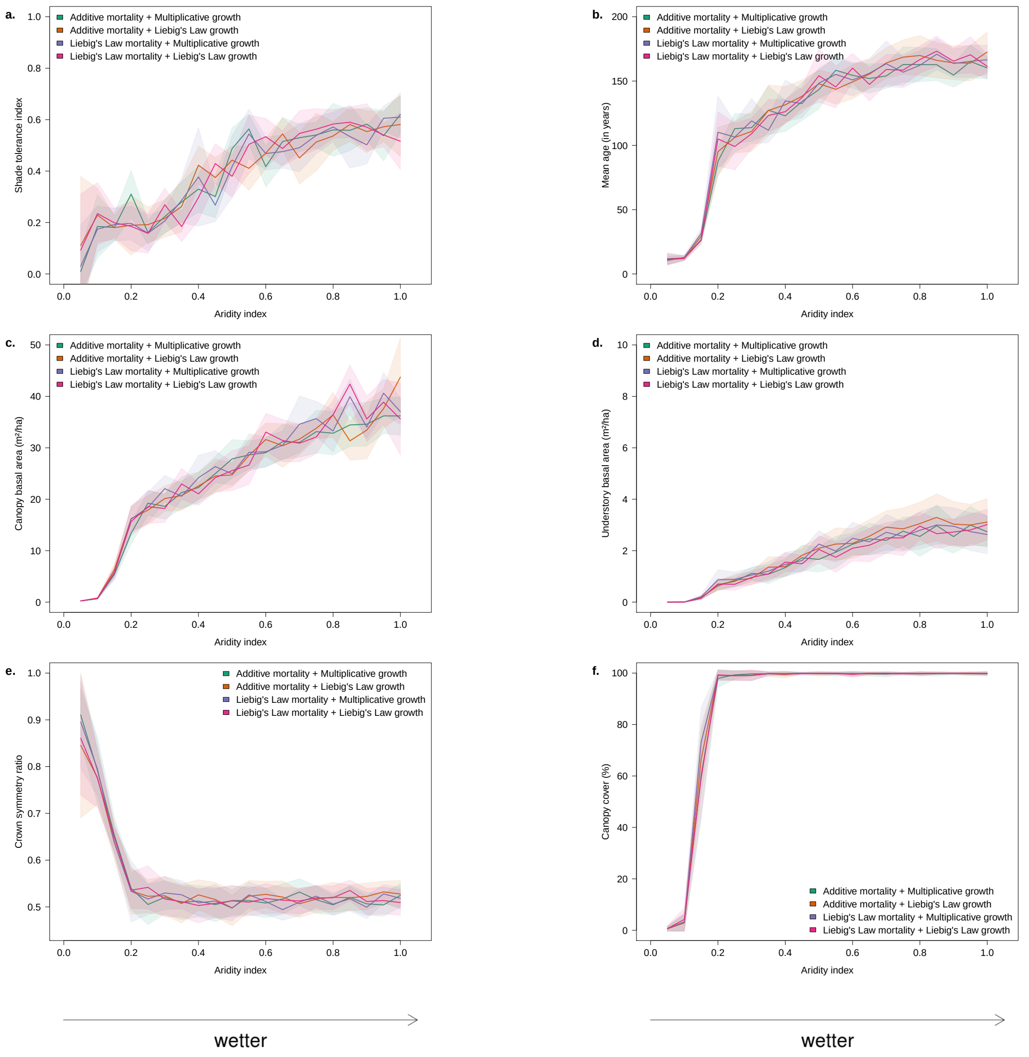

Figure S.17: Stand characteristics at equilibrium for different soil aridity indices, from 0 (xeric) up to 1 (mesic). Similar to Fig. 6 in main text, except that the maximal mortality level for water-limited trees was 40%.

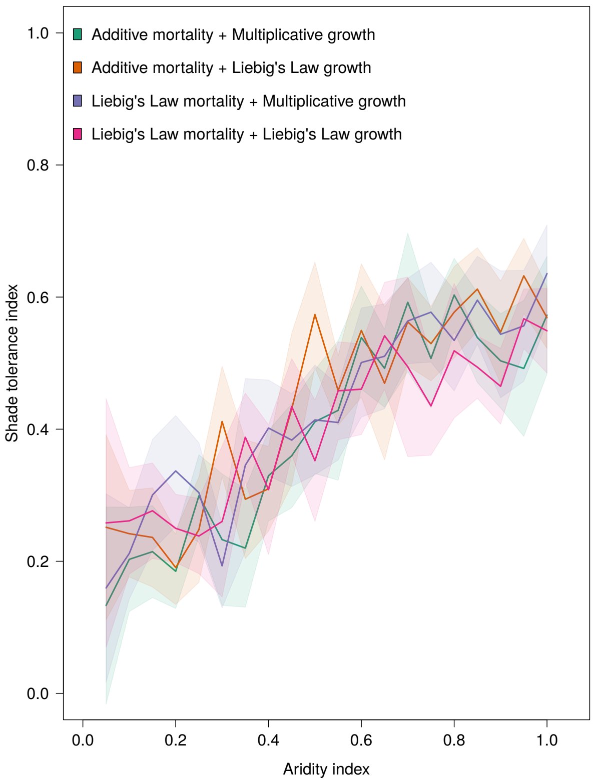

Figure S.18: Demonstration of the robustness of the model predictions with respect to variation of species specific parameters. Shade tolerance index at equilibrium for different soil aridity indices, from 0 (xeric) up to 1 (mesic) for simulations with intermediate species parameters. The increase in the shade tolerance index along the aridity gradient is similar to the pattern obtained with prototype species.
